# Dietary Toll-Like Receptor Stimulants Promote Hepatic Inflammation and Impair Reverse Cholesterol Transport in Mice via Macrophage-Dependent Interleukin-1 Production

**DOI:** 10.3389/fimmu.2019.01404

**Published:** 2019-06-20

**Authors:** Tola A. Faraj, Cordula Stover, Clett Erridge

**Affiliations:** ^1^Department of Cardiovascular Sciences, University of Leicester, Glenfield Hospital, Leicester, United Kingdom; ^2^Department of Pharmacognosy, Hawler Medical University, Erbil, Iraq; ^3^Department of Infection, Immunity and Inflammation, University of Leicester, Leicester, United Kingdom; ^4^School of Life Sciences, Anglia Ruskin University, Cambridge, United Kingdom

**Keywords:** microbiota, inflammation, innate immunity, cholesterol metabolism, cardiovascular disease, diet

## Abstract

**Background:** The mechanisms connecting dietary intake of processed foods with systemic inflammatory markers and cardiovascular risk remain poorly defined. We sought to compare the abundance of pro-inflammatory stimulants of innate immune receptors in processed foods with those produced by the murine ileal and caecal microbiota, and to explore the impact of their ingestion on systemic inflammation and lipid metabolism *in vivo*.

**Methods and results:** Calibrated receptor-dependent reporter assays revealed that many processed foods, particularly those based on minced meats, contain pro-inflammatory stimulants of Toll-like receptor (TLR)-2 and TLR4 at concentrations which greatly exceed those produced by the endogenous murine ileal microbiota. Chronic dietary supplementation with these stimulants, at concentrations relevant to those measured in the Western diet, promoted hepatic inflammation and reduced several markers of reverse cholesterol transport (RCT) in mice. Hepatocytes were found to be insensitive to TLR2- and TLR4-stimulants directly, but their secretion of functional cholesterol acceptors was impaired by interleukin (IL)-1β released by TLR-responsive hepatic macrophages. Hepatic macrophage priming by high-fat diet enhanced the impairment of RCT by ingested endotoxin, and this was reversed by macrophage depletion via clodronate liposome treatment, or genetic deficiency in the IL-1 receptor.

**Conclusion:** These findings reveal an unexpected mechanism connecting processed food consumption with cardiovascular risk factors, and introduce the food microbiota as a potential target for therapeutic regulation of lipid metabolism.

## Introduction

Atherosclerosis is a chronic inflammatory disease of the arteries that represents the root cause of the majority of cardiovascular diseases (1). Diet, inflammation and lipid metabolism are well-established risk factors for this disease. However, potential mechanisms linking these factors remain to be clearly defined. In particular, the mechanisms by which adherence to the Western dietary pattern increases circulating inflammatory markers and risk of coronary artery disease (CAD), relative to prudent diets, remain poorly understood (2, 3). Evidence from murine models of diet-induced atherosclerosis suggests that inflammation triggered via the innate immune receptors Toll-like receptor (TLR)-2 and TLR4 is likely to be involved (4, 5). However, the stimuli responsible for the triggering of atherogenic TLR-signaling, and how these may relate to diet, lipid metabolism or CAD risk, remain to be established.

In earlier work, we found that many processed foods trigger inflammatory signaling in human monocytes *in vitro* in a manner dependent on food content of the canonical ligands of TLR2 and TLR4 (namely bacterial lipopeptides and lipopolysaccharides, LPS), which are introduced into foods by common, non-pathogenic food spoilage bacteria (6, 7). Human feeding studies then showed that the consumption of diets rich in these TLR-stimulants increased several markers of cardiovascular risk, including low-density lipoprotein (LDL)-cholesterol, and inflammatory markers, relative to a low TLR-stimulant run-in diet (8). Likewise, nutritionally identical meals differing only in content of bacterial molecules had opposing effects on the post-prandial induction of leukocyte inflammatory markers (8).

However, these findings were difficult to explain, since the human commensal microbiota comprises ~100 trillion bacteria, and a burden of pro-inflammatory pathogen-associated molecular patterns (PAMPs) that presumably far exceeds that contained in the diet (9, 10). Enterocytes are also reported to be largely unresponsive to apical TLR-stimulants, and when their intracellular or basolateral TLRs are triggered, a barrier enhancing effect, rather than an inflammatory response, is typically induced (11, 12). Systemic absorption of appreciable quantities of dietary PAMPs is also considered to be unlikely, as earlier studies demonstrated extremely low or undetectable uptake of ingested endotoxin to serum, and no obvious pathology in LPS feeding studies (13, 14). In light of these observations, which are presumed to reflect adaptations which limit host inflammatory responses to the commensal microbiota, it is not clear how dietary TLR-stimulants, ingested at doses far lower than thought to be present in the intestine, could alter systemic inflammatory tone, or lipoprotein metabolism.

We therefore aimed to revisit these assumptions using a variety of approaches to PAMP quantitation and tracking. Potential mechanisms connecting dietary PAMP intake with systemic inflammatory signaling and lipid metabolism were then explored using genetic and pharmacological approaches in murine models of dietary PAMP supplementation.

## Methods

### Mice

Wild-type (WT) C57BL/6, and *Il1r1*^−/−^ mice fully backcrossed onto the C57BL/6 line, were purchased from Charles River (UK) and used after 7 days acclimatization at the University of Leicester specific pathogen free (SPF) animal housing facility, or bred within the facility for use in experiments from 7 weeks of age. Genotype was confirmed by PCR. Male mice were used for all experiments to limit the potential impact of estrous cycle on inflammatory markers. Diets used were normal (low-fat) mouse chow (TestDiet, 5LF2), high fat diet (TestDiet, 5TJN) and high cholesterol diet (TestDiet, 5TJT). Mice were randomly allocated to control or PAMP treatment by technicians blinded to experimental outcomes, and littermates were generally split to receive both treatments. All mouse experiments were conducted according to Home Office guidelines and with institutional and Home Office approval (PPL60/4332).

### Human Subjects

Peripheral blood mononuclear cells (PBMC) were prepared from venous blood collected by venepuncture from healthy human volunteers with informed, written consent (age 22–35 years, *n* = 7, all male) according to the guidelines laid down in the Declaration of Helsinki and with approval from the University of Leicester College of Medicine Research Ethics Committee. Exclusion criteria included self-reporting of a previous diagnosis of any chronic inflammatory disease (such as arthritis or inflammatory bowel disease), infection within the previous 4 weeks or use of any medication other than oral contraceptives within the last week.

### Preparation of Food and Microbiota Extracts

Foods from three major categories previously identified to be at high risk of containing elevated levels of PAMPs [minced meat, diced onion, and chocolate containing products (6)] were purchased from local supermarkets. Extracts were prepared by homogenizing 25 g of each product in 250 ml phosphate buffered saline (PBS). The samples were then clarified by centrifugation (13,000 g for 5 min) and the resulting supernatant was filter-sterilized (0.22 μm, low-protein binding filters, Acrodisc). This step was taken to prevent the growth of microbes during tissue culture, which would interfere with TLR-stimulant quantitation, and also to represent the free, soluble PAMPs present in each sample, since these are more likely to be absorbed from the gut lumen than PAMPs which remain attached to or within bacterial cells. Samples were stored at −20°C before batch assay for TLR-stimulant content. Human and murine stool samples were collected with informed consent and institutional ethical approval and were processed in the same way.

### Quantification of TLR-Stimulants

We focused specifically on quantitation of stimulants of TLR2 and TLR4 in this project because inflammatory cytokine production by food extracts was found to be dependent predominantly on signaling via these two receptors, and because both receptors play key roles in murine atherogenesis (4, 5). TLR-stimulants were quantified in aqueous extracts of food, stool or ileal content samples using a HEK-293 TLR-transfection assay as described previously (6). Briefly, HEK-293 cells cultured in 96 well plates were transfected with plasmids coding for the following genes: human (h)TLR2, hTLR4 (co-expressing hMD-2) or hTLR5 (each 30 ng, Invivogen), hCD14 (30 ng), thymidine-kinase promoter driven renilla reporter (internal transfection efficiency control, 10 ng) and NF-κB-sensitive luciferase-reporter (10 ng). Cells were grown for 3 days post transfection prior to 18 h challenge with extracts diluted 1:10 in Dulbecco's Modified Eagles Medium (DMEM) / 1% Fetal Calf Serum (FCS). Standard curves were prepared in all sample measurement plates using serial dilutions of Pam_3_CSK_4_, *E. coli* LPS or *S. typhimurium* flagellin. NF-κB reporter expression was calculated as fold induction relative to cells cultured in medium alone, and compared with the standard curve to yield the biological activities of each type of TLR-stimulant in the samples, relative to the respective standard. As transfected cells were sensitive to a minimum of 0.1 ng/ml LPS or Pam_3_CSK_4_, the minimum concentration of food-borne PAMPs detectable by the assay was 10 ng LPS- or Pam_3_CSK_4_-equivalents per g food.

### Dietary PAMP Supplementation and Oral Gavage Experiments

**Figure 1:**
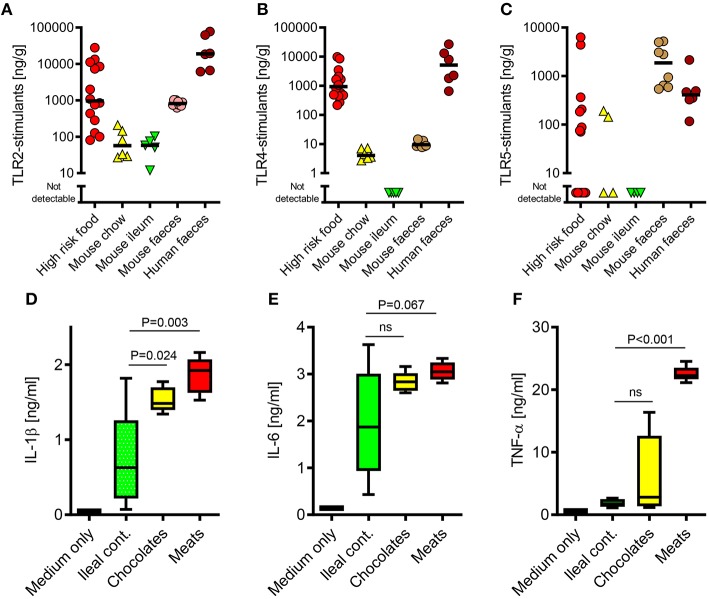
The TLR-stimulant content of some processed foods can greatly exceed that of the normal mouse ileal microbiota. **(A–C)** Soluble TLR2, TLR4, and TLR5-stimulants were quantified in processed foods (*n* = 14), mouse chow (*n* = 6), mouse ileal ingesta (*n* = 5), mouse feces (*n* = 10) and human feces (*n* = 6) using HEK-293-TLR transfectants. **(D-F)** Pro-inflammatory cytokine production by primary human monocytes (*n* = 3 donors) exposed to extracts of mouse ileal ingesta, minced meat or chocolate (*n* = 5 extracts of each type), diluted 1:10 (wt/vol) in culture medium. *P*-values vs. ileal contents, ANOVA with Tukey's test.

For chronic PAMP treatment, WT or *Il1r1*^−/−^ mice were given *ad libitum* normal chow and drinking water which was unmodified (control group), or supplemented with 100 μg/ml *E. coli* O111:B4 LPS, 1 μg/ml Pam_3_CSK_4_, and 1 μg/ml iEDAP (D-gamma-Glu-meso-diaminopimelic acid, a component of peptidoglycan) (PAMP group), for 8 weeks (WT mice). The chosen TLR-stimulant concentrations are comparable with the higher range of concentrations measured in foods (up to 28 μg Pam_3_CSK_4_-equivalent TLR2-stimulants per gram of minced pork, and up to 10 μg *E. coli* LPS-equivalent TLR4-stimulants per gram of diced onion, Figures 1A–C), when accounting for the ~250-fold reduced responsiveness of mice to endotoxin compared to man (15). In experiments involving acute oral PAMP challenge, mice were orally gavaged with 200 μl of saline alone or containing 1 mg Pam_3_CSK_4_, 1 mg iEDAP, or 2 mg *E. coli* LPS, as described in figure legends.

### Cells and Reagents

The PRR-stimulants Pam3CSK4, iEDAP and *Salmonella typhimurium* flagellin were from Invivogen. *E. coli* O111:B4 LPS was from Sigma. Oxidized palmitoyl arachidonyl phosphatidyl choline (OxPAPC) was prepared by air oxidation of native PAPC (Sigma) as described previously (16). Briefly, 1 mg of PAPC in chloroform was evaporated to dryness under nitrogen in a glass tube. The lipid film was then exposed to air in darkness for 72 h before resuspension to 2 mg/ml in chloroform and storage under nitrogen at −80°C. Aliquots of stock OxPAPC were dried under nitrogen in microtubes immediately before use and vortexed into pre-warmed tissue culture medium for 30 s before addition to cells. Human embryonic kidney (HEK)-293 cells (ECACC 85120602) and HepG2 cells (ECACC 85011430) were cultured in DMEM with 10% fetal calf serum (FCS) (Sigma). The murine macrophage cell-lines RAW 264.7 (ECACC 91062702) and J774A.1 (ECACC 91051511) were cultured in RPMI with 10% FCS. Testing of cell-lines for mycoplasma contamination by PCR revealed no such infections, and cells were routinely cultured with Plasmocin (Invivogen) to prevent growth of mycoplasma. Recombinant IL-1β, IL-6, and TNF-α were from Peprotech EC Ltd. Neutralizing anti-IL-1β antibody was from R&D Systems (AF-201-NA, used at 2 μg/ml).

### Human Monocyte Culture and Challenge

Peripheral blood mononuclear cells (PBMC) were prepared by density gradient centrifugation at 800 g for 25 min using Histopaque-1077 (Sigma). Recovered cells were washed twice in PBS, resuspended in RPMI with 10% FCS and plated in 96-well plates at 4 × 10^5^ cells per well. Monocytes were prepared from PBMC by plastic adherence (1 h at 37°C), followed by gentle washing to remove non-adherent cells. Remaining monocytes, which were of ~85–90% purity as measured by CD14 staining by flow cytometry, were then challenged by adding a 1:20 dilution of each filter-sterilized food extract in tissue culture medium. For inhibition of TLR2 and TLR4, OxPAPC was used at 30 μg/ml, as this has been shown previously to result in specific inhibition of TLR2 and TLR4 signaling, but not signaling initiated via other TLRs or cytokine receptors (17, 18). Production of TNF-α was measured at 4 h, and IL-1β and IL-6 at 24 h, by ELISA (R&D).

### Bacterial Culture and 16S Ribosomal (r)RNA Gene qPCR

TLR-stimulant secretion was measured in conditioned media of the following members of the food or human commensal microbiota: *Lactobacillus plantarum* (NCIMB-6376), *Escherichia coli* (NCTC-13114), *Xanthomonas campestris* (8004), *Pseudomonas fluorescens* (SBW25), *Pseudomonas fluorescens* (WCS365), *Pectobacterium atrosepticum* (ECG12), and *Erwinia chrysanthemi* (EC16) were kindly provided by Dr. Rob Jackson, University of Reading. *Bacteroides fragilis* (NCTC-9343) and *Bifidobacterium bifidum* cells and supernatant were kind gifts of Professor Ian Poxton (University of Edinburgh). *Streptococcus pneumoniae, Staphylococcus aureus, Pseudomonas diminuta, Enterococcus faecalis, Pseudomonas aeruginosa, Salmonella typhimurium* were University of Strathclyde teaching laboratory reference strains kindly provided by David McNeill (University of Strathclyde). Each strain was grown in respective standard broths and conditions to absorbance at 600 nm of 1.0, and centrifuged to pellet bacterial cells (13,000 g for 5 min). The conditioned medium was then filter-sterilized and stored at −20°C prior to assay. For 16S ribsosomal RNA (rRNA) gene qPCR, DNA was extracted from food and stool samples using the Qiamp Stool DNA extraction kit (Qiagen). Total historical bacterial growth was measured by quantitative PCR using the universal 16S rRNA gene primers 926F (AAACTCAAAKGAATTGACGG) and 1062R (CTCACRRCACGAGCTGAC), as described previously (19). The proteobacterial 16S rRNA gene specific primers 1080gF (TCGTCAGCTCGTGTYGTGA) and g1202R (CGTAAGGGCCATGATG) were then used to measure the percentage of this total which was derived from growth of proteobacteria. Samples were quantitated relative to an internal standard (*E. coli* NCTC-13114 genomic DNA) using a Rotorgene thermal cycler (Qiagen).

### Histology and Immunohistochemistry

For histology and some immunohistochemistry experiments, mouse tissue samples were fixed in 4% paraformaldehyde overnight before processing to wax blocks. Five micrometers of sections were then subjected to haematoxylin and eosin (H&E) staining according to standard protocols. For hepatic F4/80, TLR2 and TLR4 immunofluorescence imaging, tissue samples were frozen immediately in optimal cutting temperature compound (CellPath) and stored at −80°C. Seven micrometers of sections were then prepared using a cryostat (Leica CM3050S). Antibodies used were rat-anti-mouse F4/80 (Abd Serotec, MCA497GA, 1:100) with AF488-goat-anti-rat (Invitrogen, A-11006, 1:500) or rabbit anti-rat HRP Fab'2 (Abd Serotec, STAR21B, 1:100) secondaries. Rabbit anti-mouse TLR2 (Santa-cruz, sc10739, 1:100) and rabbit anti-mouse TLR4 (Santa-cruz, sc10741, 1:100) used AF594-labeled goat anti-rabbit secondary (Invitrogen, A-11037, 1:500). Nuclei were stained using ProLong® Gold Antifade Reagent containing DAPI (Invitrogen). Histological markers of intestinal inflammation were considered to include mononuclear or polymorphonuclear cell infiltrates, or reduced mean villus height.

### ELISA and Western Blots

Serum amyloid A (SAA) was measured in mouse plasma by ELISA (Abcam). Human and mouse IL-1β, IL-6, and TNF-α levels were measured by respective DuoSet ELISA kits (R&D). Western blots were probed with antibodies targeting IL-1β (Peprotech, 500-P51, 1:1,000), ApoAI (Cell Signaling Technology, 3350S for human, 1:250, Santa-cruz sc-30089 for mouse, 1:250) or glyceraldehyde 3-phosphate dehydrogenase (GAPDH, Santa-cruz, sc-25778, 1:2,000). Serum ApoAI levels were measured by densitometry following Western blot using ImageQuant software (GE Healthcare), and normalized to total plasma protein, measured using the bicinchoninic acid assay (Pierce).

### Lipoprotein and Cholesterol Efflux Assays

For depletion of ApoB-containing lipoproteins from mouse serum samples, 10 μl of 20% polyethylene glycol (PEG) in 200 mM glycine buffer, pH 7.4, was added to 25 μl serum, incubated at room temperature for 20 min, then centrifuged at 10,000 g for 30 min to pellet complexed ApoB lipoproteins. The remaining ApoB-depleted serum was used for measurement of HDL-C (using the Amplex cholesterol assay, Invitrogen), and serum cholesterol efflux capacity, which was measured as previously described (20). Briefly, J774 macrophages were plated at 3 × 10^5^ cells per well of 24-well plates and labeled overnight with 67 kBq/mL ^3^H-cholesterol (Perkin-Elmer). After equilibration for a further 6 h in DMEM containing 14 mM HEPES, cells were cultured with 500 μl efflux medium (MEM containing 14 mM HEPES and 0.15 mM cAMP) supplemented (or not) with 14 μl ApoB-depleted serum. After 4 h, effluxed and cell-bound cholesterol was measured by scintillation. For measurement of functional cholesterol acceptors secreted by HepG2 cells, 50 μl of HepG2 cell conditioned medium was added to 450 μl of efflux medium in the J774 assay. Triglycerides and glucose levels were measured in fasted mouse serum using kits from Wako and Invitrogen, respectively.

### Measurement of LPS Translocation

LPS activity was measured in mouse plasma samples using the Pyrochrome limulus amoebocyte lysate (LAL) assay (Associates of Cape Cod, UK) after recovery of LPS bioactivity by heat treatment (70°C for 10 min). LAL-activities were determined by kinetic analysis of each reaction using a temperature controlled microplate reader (BMG Lumistar), as previously described (8). Serum LPS levels were below the limit of detection using the HEK-293 TLR4 transfection assay. In some experiments, mice were orally gavaged with 200 μl saline containing either 1 mg fluorescein isothiocyanate (FITC)-LPS (Sigma), or 15 mg FITC-dextran (MWT 4 kDa, Sigma) with 2 mg *E. coli* LPS. FITC-LPS translocation to intestinal villi was monitored at 2.5 h by fluorescence microscopy. Percentage translocation of FITC-dextran was quantified by fluorescence of plasma samples collected by cardiac puncture 1.5 h after oral gavage, in comparison with respective standard curves. For quantitation of translocation of lumenal LPS to tissues, mice were orally gavaged with 1 mg ^3^H-labeled LPS in 200 μl PBS. For ^3^H-labeling, *E. coli* cells were cultured to an optical density of 0.9 at absorbance of 600 nm in broth supplemented with 0.74 MBq ^3^H-acetate (Perkin-Elmer) per ml as previously described (21). LPS was then extracted by the MgCl_2_ precipitation method as previously described (22), yielding a specific activity of 3 kBq/mg. Organs were extracted 4 h post-gavage, and 100 mg of each tissue was homogenized in 1 ml 0.1% sodium dodecyl sulfate (SDS)/0.2M NaOH using a bead homogeniser (Precellys). Radiolabel content of tissue extracts was quantified by scintillation.

### Priming Experiments

In experiments aiming to increase absorption of LPS from the gut lumen, mice were fed HFD for 4 weeks, or orally gavaged with 2 (low dose) or 10 (high dose) mg/kg indomethacin, 24 h before oral gavage with 2 mg *E. coli* LPS or fluorescent markers as described above. In experiments aiming to increase *in vivo* sensitivity to LPS, mice were fed HFD or high cholesterol diet for 4 weeks before oral gavage with 2 mg *E. coli* LPS (23, 24). Alternatively, mice fed normal chow received intravenous injection of 200 μl normal saline containing 1 mg heat-killed (60°C, 1 h) *Propionibacterium acnes*, 7 days before oral gavage with 2 mg *E. coli* LPS.

### Clodronate Liposome Treatment

Twenty-two male C57 mice were given unrestricted access to high fat diet (5TJN) and normal drinking water for 4 weeks. All mice then received an intraperitoneal injection with 1 mg clodronate liposomes (www.clodronateliposomes.com) in 200 μl saline. 72 h later, mice were orally gavaged with 200 μl PBS alone or containing 2 mg *E. coli* LPS (*n* = 11 per group). Blood was collected by cardiac puncture 24 h after oral gavage. Liver sections were stained for F4/80 positivity to monitor effects of clodronate liposome treatment on hepatic macrophage number.

### Flow Cytometry

For mouse primary hepatic macrophage and hepatocyte flow cytometry, 10^5^ cells were labeled with 4 μg/ml of isotype control antibody (rat IgG2a-PE, Cambridge Bioscience 400507, 1:50), rat anti-mouse TLR2-PE (Cambridge Bioscience, 148603, 1:50) or anti-TLR4-PE (Cambridge Bioscience, 145403, 1:50). For human monocyte, PBMC and HepG2 cell-line flow cytometry, antibodies used were AF488-labeled anti-TLR2, anti-TLR4 and mouse IgG2a-K isotype control (eBioscience, 53-9922-41, 1:20, 53-9917-41, 1:20 & 53-4724-80, 1:50). Data were acquired using a Gallios cytometer (Beckman Coulter).

### Hepatocyte and Hepatic Macrophage Isolation

Mouse primary hepatocytes and hepatic macrophages were prepared by the three-step perfusion method as described previously, (25) with the following modifications. Livers were perfused by cannula via the vena cava and drained via the portal vein with the following buffers in sequence: (i) 25 ml clearing medium (Hanks balanced salt solution with 0.5 mM EGTA, pH 7.4), (ii) 10 ml PBS, (iii) 25 ml digestion buffer [DMEM with HEPES and 0.5 mg/ml type IV collagenase (Sigma)]. A perfusion pump was used to regulate flow to ~4 ml/minute and a water bath jacket was used to maintain buffers at 37°C during perfusion. Once the liver had swollen indicating successful digestion of collagen, it was carefully excised, the gall bladder removed, and cells extracted by gentle agitation in isolation medium (ice cold DMEM with HEPES, penicillin, streptomycin and 10% fetal calf serum). Hepatocytes and hepatic macrophages were then isolated by the differential density method as described previously. (25) Briefly, mixed liver cell suspensions were centrifuged at 4°C for 2 min at 50 g. The hepatocyte-rich pellet was resuspended in 25 ml of cold isolation medium and the centrifugation procedure was repeated twice more (2 min, 50 g washes) to further purify the hepatocytes. Hepatocytes were then plated at 100,000 viable cells per well of collagen coated 24-well plates, allowed to attach for 1 h at 37°C, and then cultured in growth medium (DMEM with 10% FCS) overnight before use in experiments. Macrophages were collected from the supernatant of this first wash, purified by further centrifugations at 50 g, then plated at 80,000 viable cells per well of non-coated 24-well plates, allowed to adhere without collagen for 16 min at 37°C, then washed to remove residual hepatocytes, sinusoidal and endothelial cells. Cell purity was measured by flow cytometry for the TLR2 antigen, which was expressed by >90% of isolated macrophages, but undetectable on hepatocytes isolated as above. For mouse liver slice culture, livers were excised immediately after euthanasia and tissue cylinders were prepared using a sterilized 1 cm diameter metal punch. Circular sections of 300 μm thickness were then prepared in ice-cold RPMI medium using a Microslicer DTK-1000 vibratome. Sections were cultured in independent wells of 24-well plates and challenged with PAMPs or cytokines for 24 h before measurement of cytokine production by ELISA. Because IL-1β production was below the limit of detection in this system, liver slice culture was not pursued in further experiments.

### Statistical Analyses

In experiments where more than two groups were compared, one way analysis of variance (ANOVA) was performed using Tukey's or Dunnett's post-test. In experiments where two groups were compared, unpaired, two-tailed Student's *T*-tests, or the Mann–Whitney *U*-test, were used. Food and intestinal PAMP concentrations were log_10_-transformed before analysis. Differences were assumed to be significant at *P* < 0.05. Each point plotted on categorical scatter plots corresponds to measurements taken from independent mice. Biological rather than technical replicates are shown throughout. Error bars shown on bar charts are standard error of the mean (SEM). Data were analyzed using GraphPad Prism version 7.0.

## Results

### The PAMP Content of Some Commonly Consumed Foods can Greatly Exceed That of the Endogenous Murine Ileal Microbiota

**Figure 2:**
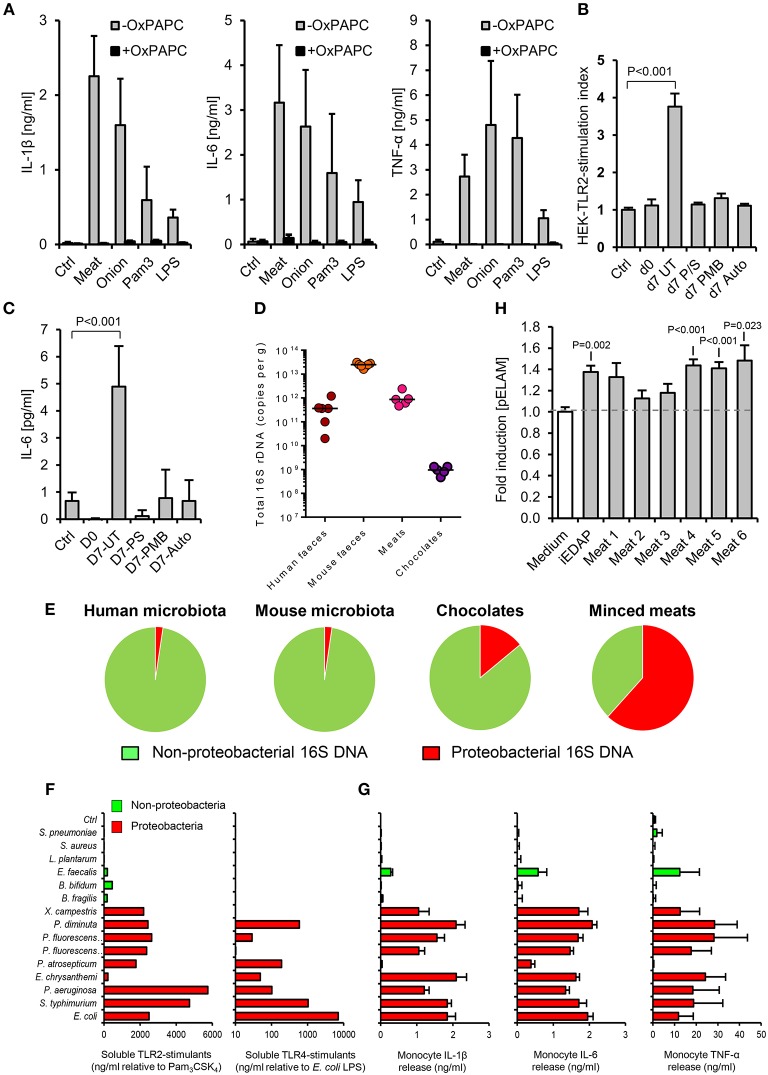
The accumulation of TLR-stimulants in processed meat is dependent on the food microbiota. **(A)** Human primary monocytes were treated with filter-sterilized extracts of minced meat or diced onion, 1 ng/ml *E. coli* LPS or 100 ng/ml Pam_3_CSK_4_, with or without oxidized palmitoyl arachidonly phosphatidyl choline (OxPAPC, an inhibitor of TLR2 and TLR4, *n* = 4 volunteers). **(B)** NF-κB activation in HEK-293-TLR2 transfectants challenged with filter-sterilized extracts of minced meat samples when fresh, or at the ‘best before date’ (d7), with or without treatment with the antibiotics penicillin and streptomycin (P/S), or polymyxin-B (PMB, which specifically targets Gram-negative bacteria), or autoclaving before storage. **(C)** Cytokine responses of human primary monocytes to the same meat extracts (*n* = 4 per group). **(D)** Total 16S rDNA in stool and food samples measured by qPCR (a culture-independent measure of historical bacterial activity). **(E)** Proportion of proteobacterial 16S rDNA in human and mouse fecal samples and meat and chocolate samples (*n* = 5–7). **(F)** Relative biological activities of soluble TLR2- and TLR4-stimulants in filter-sterilized conditioned media of representative proteobacterial and non-proteobacterial organisms of the food and commensal microbiota (grown to A600 = 1.0), measured using HEK-293-TLR transfectants calibrated with Pam_3_CSK_4_ and *E. coli* LPS, respectively. **(G)** Inflammatory cytokine production by primary human monocytes cultured with 1:1,000 dilutions of the same conditioned media. **(H)** NF-κB reporter responses in HEK-293 cells transfected with NOD-1 and filter sterilized homogenates of minced meats. Error bars shown are SEM. *P*-values vs. control condition (medium alone), ANOVA with Dunnett's test.

Because the commensal microbiota is not evenly distributed along the gastrointestinal tract (9), we first sought to compare the relative biological activities of soluble stimulants of TLR2, TLR4, & TLR5 in the ileal contents of healthy mice, with those of murine and human stool samples, using a calibrated HEK-293 TLR-transfection assay (26). As expected, these stimulants were much more abundant in stool samples than in ileal contents (Figures 1A–C). However, a screen of a selection of foods previously identified to be at high risk of PAMP-contamination [minced meats, diced onion, and chocolates (6)], revealed that many of these contained TLR-stimulants at levels which were 2–3 orders of magnitude higher than normally present in the ileum. Filter-sterilized homogenates of these foods were also more potent inducers of pro-inflammatory cytokine production than murine ileal contents (Figures 1D–F), and these responses were blocked completely by OxPAPC, a specific inhibitor of TLR2 and TLR4 (Figure 2A) (17). Antibiotic treatment, or autoclaving, prior to storage of minced meats also prevented the accumulation of TLR2-stimulants in these foods, along with their capacity to induce inflammatory cytokine production, confirming that TLR-stimulants accumulate in these foods through the activity of food-borne bacteria (Figures 2B,C).

We next explored how such foods may contain similar TLR-stimulant content to the murine fecal microbiota, despite containing lower levels of total bacterial 16S ribosome gene DNA (rDNA) (Figure 2D). Because earlier work has identified proteobacteria as key inducers of inflammatory signaling in the human intestinal microbiota and during food spoilage (7, 26–28), we quantified their historical activity in samples using viability-independent qPCR. Meat and chocolate samples contained a much higher proportion of proteobacterial 16S rDNA relative to total bacterial 16S rDNA (61.9 and 14.2%), compared to mouse or human fecal microbiota samples (2.3 and 2.4%, Figure 2E). Conditioned media of selected proteobacteria also contained far more abundant soluble TLR2 and/or TLR4 stimulants than those of representative non-proteobacterial food and commensal organisms, further supporting the notion that this group may play a key role in determining the pro-inflammatory potential of extracts (Figures 2F,G). Together, these findings indicate that soluble stimulants of TLR2 and TLR4 are normally present at very low concentrations in the healthy ileum, but can be much more abundant in some processed foods, most likely due mainly to the historical activity of proteobacteria.

### Dietary PAMP Intake Promotes Hepatic Inflammation and Impaired RCT

**Figure 3:**
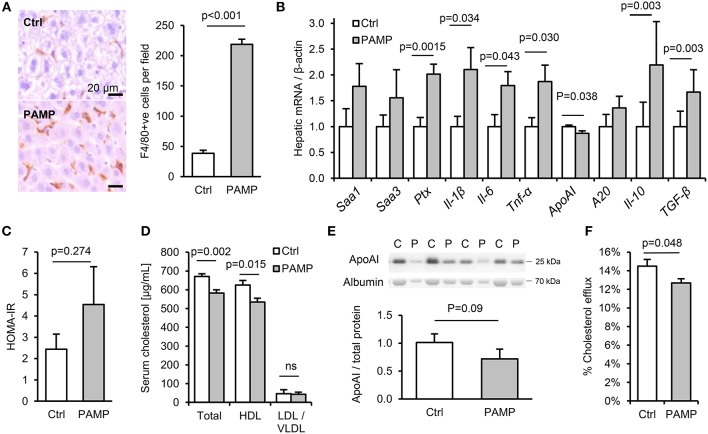
Chronic dietary PAMP intake induces hepatic inflammation and impairs serum cholesterol efflux capacity. WT mice (*n* = 8/gp) were given normal chow and drinking water supplemented (PAMP) or not (Ctrl), with *E. coli* LPS, Pam_3_CSK_4_ and D-gamma-Glu-meso-diaminopimelic acid (iEDAP, a component of peptidoglycan which stimulates NOD1), for 8 weeks. **(A,B)** Hepatic F4/80^+^ cells per field and acute phase response (APR) mRNA. **(C–F)** Serum homeostatic model assessment of insulin resistance (HOMA-IR) and RCT mediators. *P*-values vs. control condition, Student's *T*-test or ANOVA with Dunnett's test.

To explore the potential impact of chronic intake of dietary PAMPs on inflammatory and metabolic markers, C57Bl/6 mice were fed normal chow for 10 weeks with or without supplementation of drinking water with stimulants of TLR2, TLR4 and nucleotide-binding oligomerization domain-containing protein-1 (since NOD1-stimulants were also detected in minced meats, Figure 2H). The chosen concentration of lipopeptide was comparable with that measured in high-PAMP foods (6, 7), as was that of LPS when accounting for the ~250-fold reduced responsiveness of mice to endotoxin compared to man (15). Chronic dietary intake of these PAMPs had no effect on weight gain, hepatic steatosis or a variety of inflammatory and regulatory cytokines, key markers of T-cell subset abundance, villus height or Paneth cell degranulation in ileum, or histological evidence of inflammation in stomach, ileum or colon (Figures S1A–F, S2A–K). Likewise, although PAMP treatment increased macrophage markers in adipose tissue (*Cd68* mRNA and F4/80 protein), there was no significant increase in expression of inflammatory cytokines (*Il-1*β, *Il-6*, or *Tnf*-α) in fat (Figures S2E,H). However, a number of markers of inflammation and the acute phase response (APR) were upregulated in livers of PAMP-fed mice compared to controls, consistent with a ~6-fold increase in hepatic F4/80^+^ macrophages (Figures 3A,B). Although PAMP treatment did not significantly alter homeostatic model assessment of insulin resistance (HOMA-IR) or serum triglycerides (Figure S2D; Figure 3C), total cholesterol was lower in serum of treated mice, driven by significant reductions in HDL-C (Figure 3D). PAMP treatment also reduced hepatic expression of *ApoAI* mRNA and serum ApoAI protein (Figures 3B,E), and significantly impaired the capacity of ApoB-depleted serum to accept cholesterol effluxed from macrophages—all key markers of the RCT pathway (Figure 3F).

### A Far Greater Proportion of Ingested Endotoxin Is Absorbed Than Is Detected by the Limulus Assay

**Figure 4:**
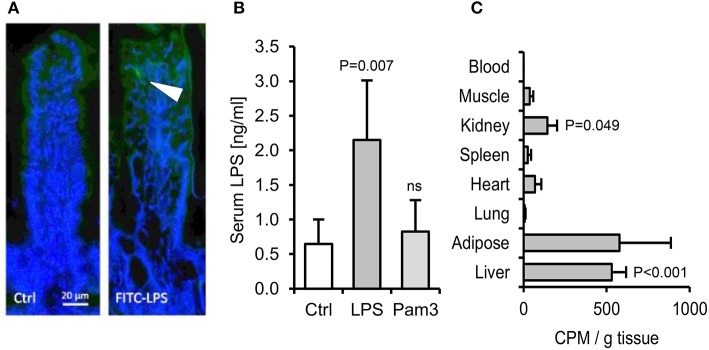
The absorption of ingested LPS is greatly underestimated by limulus and fluorescent assays for LPS uptake. **(A)** Immunofluorescence of ileal villi 90 min after gavage with saline (Ctrl) or FITC-LPS (arrow, green). **(B)** Serum LPS 2.5 h after oral gavage with saline alone, Pam_3_CSK_4_ or *E. coli* LPS, as measured using the LAL assay (*n* = 4/gp). **(C)** Radiolabel in mouse tissues 4 h after oral gavage with 1 mg ^3^H-labeled LPS (*n* = 4/gp), measured in CPM (counts per minute). *P*-values vs. Ctrl (or blood levels of radiolabel for panel **C**), ANOVA with Dunnett's test.

**Figure 5:**
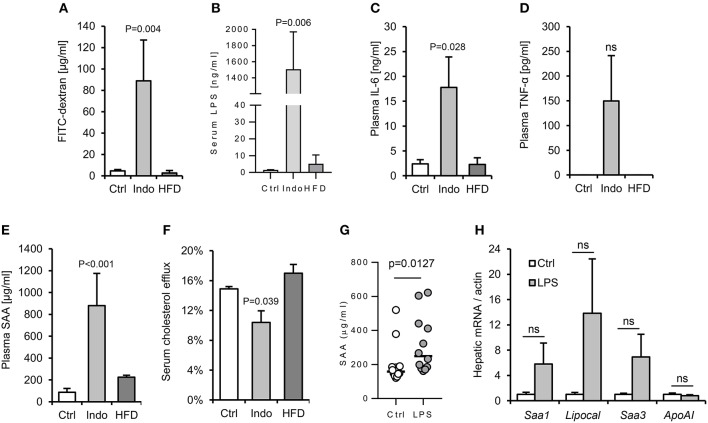
Increased gut barrier permeability is insufficient to reproducibly increase hepatic APR markers in response to a single oral gavage with LPS. Two different approaches were taken with to increase gut permeability and enhance translocation of LPS from gut lumen to serum: oral pre-treatment with 10 mg/kg indomethacin (31) and 4 weeks high fat diet (HFD). (32) WT mice (*n* = 4/gp) were then orally gavaged with 15 mg FITC-dextran and 2 mg *E. coli* LPS in 200 μl saline. Indomethacin, but not HFD, increased gut permeability to LPS and FITC-dextran, although with excessive background systemic inflammation **(A–F)**. Lower dose indomethacin (2 mg/kg, *n* = 12/gp), resulted in a ~14-fold increase in circulating LPS 90 min post LPS-gavage, and enabled a plasma serum amyloid A (SAA) response to LPS orally delivered later **(G)**, but hepatic acute phase response (APR) mRNA markers were not reproducibly increased **(H)**. Error bars shown are SEM. *P*-values vs. control condition, ANOVA with Dunnett's test.

We next aimed to quantify the fraction of ingested LPS that may translocate to blood. Orally gavaged FITC-labeled LPS was detectable within occasional villi post-gavage (Figure 4A), but fluorescence was undetectable in blood, liver or spleen. Measurements made using the more sensitive limulus assay, which is traditionally used for this purpose (29), also suggested that only a very small proportion of the dose is absorbed to blood (~0.00004%, assuming a mouse blood volume of 2 ml and 1 mg LPS ingested, Figure 4B). However, because the capacity of LPS to stimulate the limulus assay is rapidly diminished by blood proteins *in vivo* (30), we sought to measure tissue uptake of orally delivered ^3^H-labeled LPS. This approach revealed that although little or no radiolabel was detected in heart, muscle, lung or blood post-gavage (suggesting rapid clearance of absorbed LPS), there was significant accumulation (~0.4% of the dose) of ^3^H-LPS in liver (Figure 4C). This raised the possibility that sufficient LPS may be absorbed from the diet to trigger the hepatic inflammation observed in earlier experiments. However, we found that oral delivery of the tested PAMPs, alone or in combination, did not reproducibly induce dyslipidaemia or hepatic inflammatory markers within 24 h in unprimed mice (Figures S3A–G). Attempts to increase LPS absorption using indomethacin, an established enhancer of gut permeability (31), resulted in markedly elevated serum LPS levels post-LPS-gavage, but hepatic APR markers were still not reproducibly elevated, suggesting that the unprimed mouse liver displays low responsiveness to relatively high levels of circulating LPS in health (Figures 5A–H).

### Hepatic Macrophages Mediate the APR and Cholesterol Efflux Responses to Dietary Endotoxin

**Figure 6:**
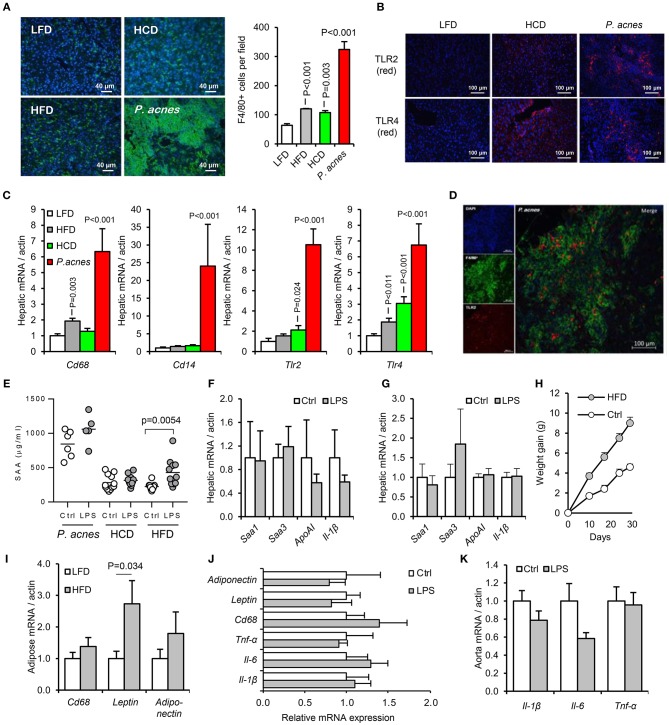
Development of an acute murine model of oral-LPS induced APR and dyslipidaemia. Three methods previously reported to increase the sensitivity of mice to circulating LPS were tested, (i) intraperitoneal administration of heat-killed *Propionibacterium acnes* (*n* = 6 per group), (ii) 4 weeks priming with high fat diet (HFD, *n* = 10 per group) or (iii) 4 weeks priming with high cholesterol diet (HCD), (*n* = 12 per group) (23, 24, 33) **(A)** Hepatic F4/80^+ve^ (green) cell number was significantly elevated by all three treatments relative to control mice fed normal chow. **(B)** HCD and *P. acnes* treatment increased hepatic TLR2 and TLR4 protein expression (red) from a very low baseline. **(C)** Hepatic CD68, CD14, TLR2, and TLR4 mRNA responses to each treatment. **(D)** Representative overlay showing co-localization of TLR2 (red) and F4/80 (green) staining, suggesting predominant expression of TLR2 on hepatic macrophages. Similar results were observed for TLR4. **(E)** Circulating serum amyloid A (SAA) protein was significantly elevated by orally delivered LPS at 24 h only in HFD-primed mice—baseline SAA (a measure of the acute phase response) was excessively raised in HCD and *P. acnes* primed mice. **(F,G)** Hepatic APR markers and apolipoprotein (Apo)-AI were not significantly increased by orally delivered LPS in HCD and *P. acnes* primed mice, respectively. **(H)** Mice fed HFD gained significantly more body weight than mice fed normal chow. **(I)** 4 weeks HFD did not significantly increase CD68 mRNA in abdominal adipose tissue. Orally delivered LPS did not induce inflammatory markers in abdominal adipose tissue **(J)**, or aorta **(K)** of HFD-primed mice within 24 h. Error bars shown are SEM. *P*-values vs. control condition, ANOVA with Dunnett's test.

Our efforts to develop an acute model of oral-PAMP-induced APR for mechanistic studies therefore refocused on increasing the sensitivity of mice to circulating LPS. Three previously reported methods were employed: intraperitoneal administration of heat-killed *Propionibacterium acnes*, 4 weeks priming with high fat diet (HFD) and 4 weeks priming with high cholesterol diet (HCD) (23, 24, 33). While all three treatments increased hepatic macrophage number and expression of F4/80, TLR2, and TLR4 from a low baseline, and rendered mice responsive to orally delivered LPS within 24 h, only HFD priming yielded a suitably low background of APR markers for detection of hepatic inflammation in response to orally-delivered LPS (Figures 6A–H).

**Figure 7:**
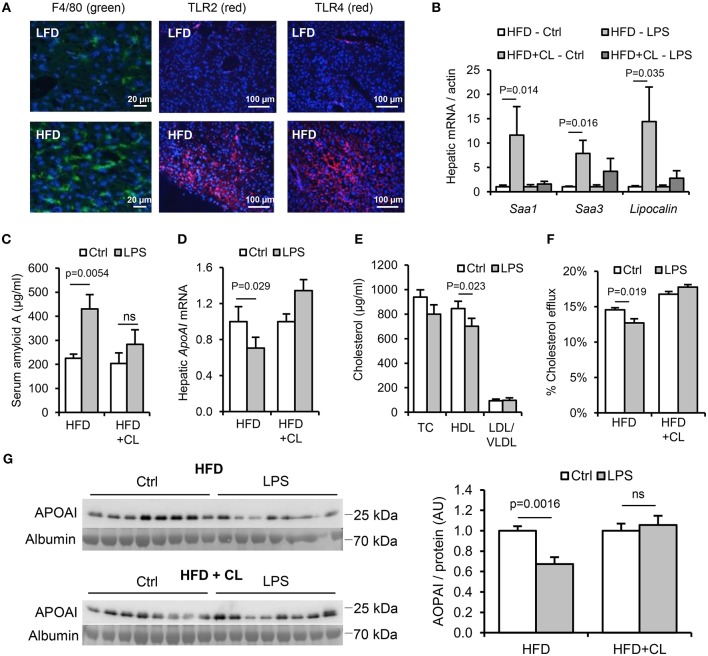
Hepatic macrophages are key mediators of the lipid and acute phase responses to ingested LPS. WT mice (*n* = 8–11/gp) were fed low-fat diet (LFD) or high-fat diet (HFD) for 4 weeks, with or without treatment with clodronate liposomes to deplete tissue macrophages. **(A)** F4/80 (green), TLR2 and TLR4 (red) immunofluorescence in livers of mice fed LFD or HFD for 4 weeks. **(B)** Hepatic acute phase response mRNA markers in saline (Ctrl) or LPS gavaged HFD-primed mice pretreated (or not) with clodronate liposomes (CL), *n* = 8–11/gp. **(C–G)** Serum amyloid A (SAA) protein, hepatic ApoAI mRNA and serum reverse cholesterol transport (RCT) markers in the same groups. *P*-values vs. control condition, ANOVA with Dunnett's test.

**Figure 8:**
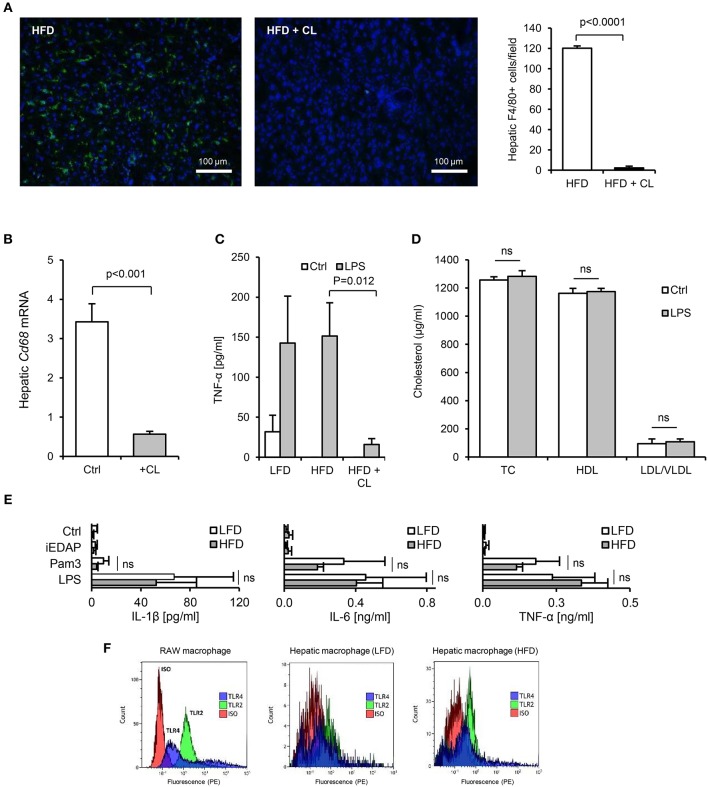
Isolated hepatic macrophages are sensitive to TLR2 and TLR4-stimulants. **(A)** Immunofluorescence (F4/80, green, DAPI, blue), showed near complete absence of F4/80^+^ macrophages in liver of mice 3 days after intraperitoneal administration of 1 mg clodronate liposomes (*n* = 8–11/gp). **(B)** Hepatic CD68 mRNA was also significantly reduced by clodronate liposome (CL) treatment. **(C)** TNF-α production from liver slices of untreated (low-fat diet, LFD), 4 week high-fat diet-primed (HFD), or 4 week HFD primed then CL treated (HFD+CL) mice (*n* = 5/gp) cultured with LPS for 24 h. **(D)** Serum cholesterol levels in mice primed for 4 weeks with HFD, then treated with CL, then orally gavaged with 200 μl saline alone (Ctrl) or 1 mg LPS, revealed no impact of orally delivered LPS on HDL-C in these mice (*n* = 11/gp). **(E)** Pro-inflammatory cytokine production by hepatic macrophages isolated from LFD- or HFD-primed mice treated with medium alone (Ctrl), Pam_3_CSK_4_ or LPS (*n* = 3–4/gp). **(F)** Flow cytometry for TLR2 and TLR4 expression by RAW macrophages (positive control cell-line), in comparison with hepatic macrophages isolated from LFD- or HFD-primed mice (representative of 3 exps). Error bars shown are SEM. Pairwise comparisons by Student's *T*-test.

In HFD-primed mice, hepatic TLR2 and TLR4 protein, APR markers and circulating SAA were significantly increased, and hepatic *ApoAI* expression, serum ApoAI, HDL-C and cholesterol efflux capacity were all significantly decreased, 24 h after oral LPS treatment (Figures 7A–G). Limulus and FITC-dextran assays showed that this increase in responsiveness was not due to effects of HFD on intestinal permeability (Figures 5A,B). However, depletion of hepatic F4/80^+^ macrophages (by ~98%, Figures 8A,B) using clodronate liposomes abolished all of the observed responses to oral LPS (Figures 7B–G, 8C,D). Oral LPS-induced APR was not likely secondary to cytokines released from macrophages in the gut, vasculature or adipose tissue, since no induction of the key APR-inducing cytokines *Il-1*β, *Il-6*, or *Tnf*-α was observed in these tissues (Figures S1D, S2H; Figures 6I–K). However, hepatic macrophages isolated from both LFD- and HFD-fed mice produced all of these cytokines in response to Pam_3_CSK_4_ or LPS stimulation *ex vivo* (Figure 8E). The responsiveness of isolated hepatic macrophages to these stimuli was not increased by HFD-priming, consistent with similar levels of expression of TLR2 and TLR4 (Figures 8E,F). Taken together, these results suggest that hepatic macrophages are key mediators of the lipid responses to orally delivered LPS, and that their increased abundance in the liver, rather than cellular responsiveness to TLR-stimulants, may be a key mechanism of HFD-induced responsiveness to orally delivered LPS.

### IL-1 Is a Key Mediator of Dietary PAMP-Mediated Downregulation of ApoAI

**Figure 9:**
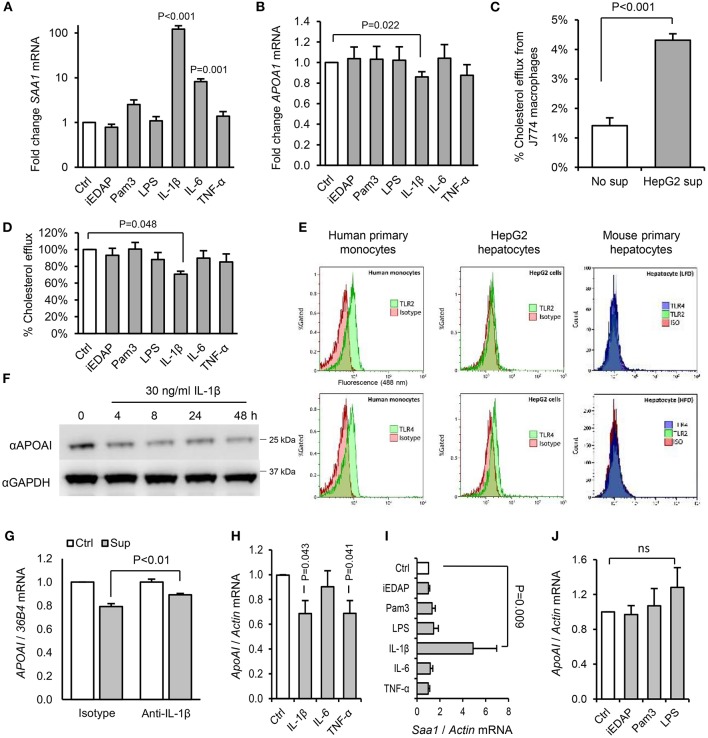
Interleukin-1β plays a key role in the downregulation of RCT mediators by hepatocytes in response to products secreted by activated hepatic macrophages. **(A,B)** Human HepG2 hepatocytes increased serum amyloid A (SAA)-1, and reduced ApoAI, mRNA expression significantly in response to IL-1β, but not stimulants of NOD1 (iEDAP), TLR2 (Pam_3_CSK_4_), or TLR4 (*E. coli* LPS). **(C)** Conditioned medium of HepG2 cells significantly increased the efflux of ^3^H-radiolabelled cholesterol from J774 macrophages. **(D)** The capacity of conditioned media of HepG2 hepatocytes to accept ^3^H-radiolabelled cholesterol effluxed from J774 macrophages was reduced significantly by IL-1β, but not by IL-6, TNF-α, or stimulants of NOD1, TLR2, or TLR4. **(E)** Flow cytometry showed abundant surface TLR2 and TLR4 on human primary monocytes, but no TLR2 and little TLR4 on HepG2 hepatocytes. Murine primary hepatocytes isolated by hepatic perfusion also showed no expression of TLR2 or TLR4, in low-fat diet (LFD) or high-fat diet (HFD)-primed mice (representative of 4 experiments). **(F)** Kinetics of ApoAI protein expression in HepG2 cells treated with IL-1β determined by Western blot. **(G)** HepG2 ApoAI mRNA response to conditioned medium of LPS-treated human monocytes with isotype-control or IL-1β neutralizing antibody (*n* = 5 exps). **(H,I)** ApoAI and SAA1 mRNA responses of primary hepatocytes (isolated from *n* = 6 LFD-fed mice) to inflammatory cytokines (20 ng/ml). **(J)** ApoAI mRNA responses of primary hepatocytes (*n* = 3 HFD-primed mice) to indicated PAMPs (100 ng/ml). Error bars shown are SEM. *P*-values vs. control condition, Student's *T*-tests or ANOVA with Dunnett's test.

Since we saw no effect of acute or chronic PAMP treatment on intestinal expression of *ApoAI* (Figure S1D), which accounts for up to 30% of circulating ApoAI protein in mice (34), we next explored the effects of PAMPs and cytokines on cholesterol acceptors expressed by hepatocytes. IL-1β, but not IL-6, TNF-α, or ligands of TLR2, TLR4, or NOD1 significantly reduced ApoAI mRNA expression in human HepG2 hepatocytes (Figures 9A,B). HepG2 cells also secrete HDL particles similar to those found in plasma (35), and we found that the capacity of their conditioned medium to accept cholesterol effluxed from macrophages was significantly reduced by IL-1β, but not by IL-6, TNF-α, or TLR-stimulants directly (Figures 9C,D). This was consistent with reduced expression of ApoAI protein by IL-1β treatment and lack of expression of TLR2 or TLR4 protein in these cells and mouse primary hepatocytes (Figures 9E,F). IL-1β neutralizing antibody also reversed the inhibition of HepG2 ApoAI expression by supernatant of LPS-stimulated human monocytes, suggesting a role for IL-1β in myeloid cell-induced hepatocyte APR (Figures 9G–J).

**Figure 10:**
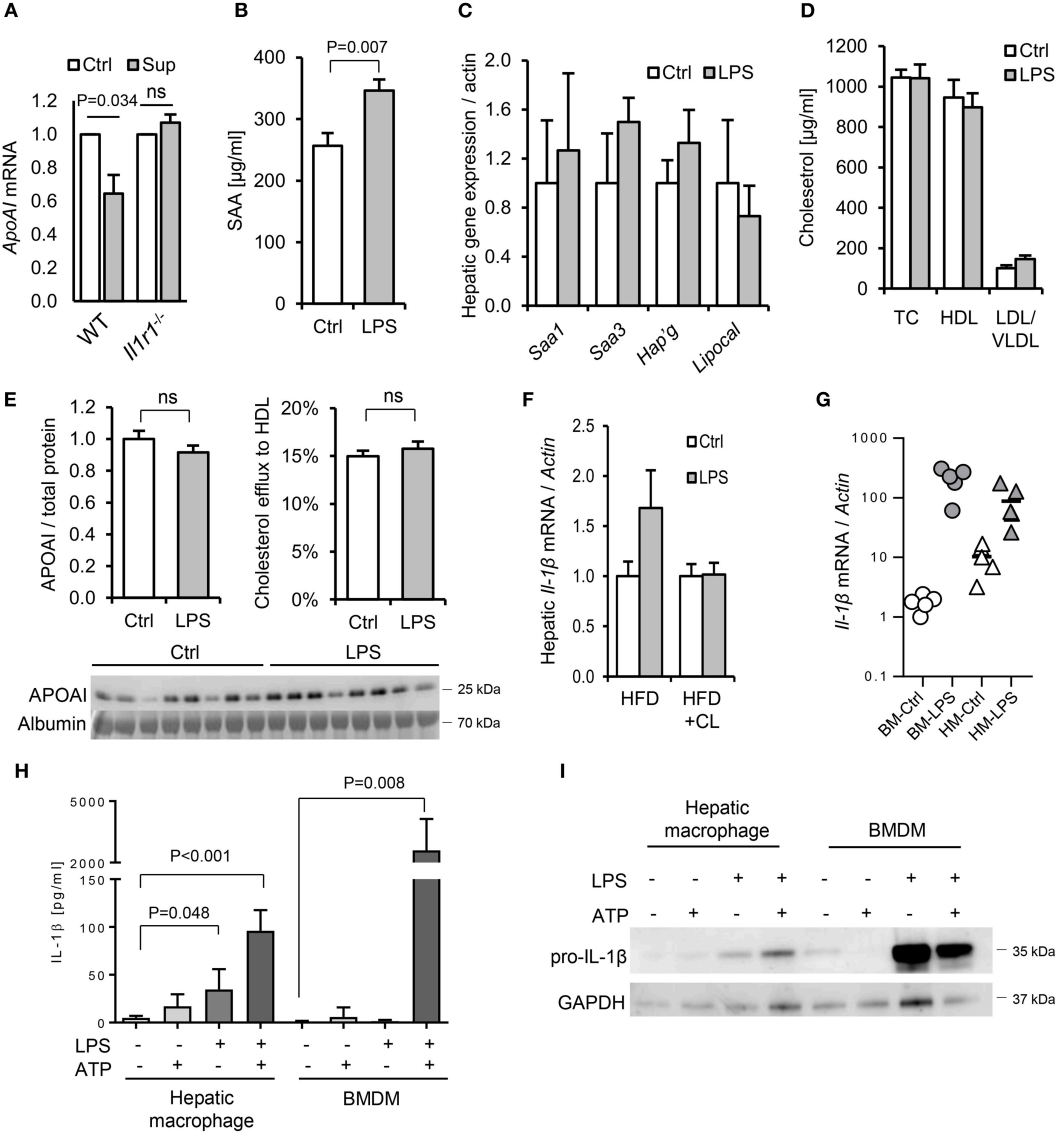
Interleukin-1 is a key mediator of the lipid and acute phase responses to ingested LPS *in vivo*. **(A)** ApoAI mRNA responses of isolated wild-type (WT) and interleukin-1 receptor deficient (*Il1r1*^−/−^) hepatocytes to conditioned medium of LPS-treated WT hepatic macrophages. **(B–D)** Plasma serum amyloid A (SAA), lipoprotein cholesterol and hepatic acute phase response (APR) mRNA responses in HFD-primed *Il1r1*^−/−^ mice 24 h after oral gavage with saline alone (Ctrl) or 1 mg *E. coli* LPS (*n* = 8, 10/gp). **(E)** ApoAI protein and efflux capacity in sera of the same mice. **(F)** Hepatic IL-1β mRNA in LPS-gavaged HFD-primed WT mice treated (or not) with clodronate liposomes (CL). **(G)** IL-1β mRNA in LPS-treated isolated primary mouse bone marrow-derived macrophages (BMDM) and hepatic macrophages (HM). **(H)** Secreted and **(I)**, cytosolic IL-1β protein production by LPS and ATP-stimulated primary mouse BMDM and hepatic macrophages (*n* = 3–5/gp). *P*-values vs. Ctrl, ANOVA with Dunnett's test.

To further explore the potential role of IL-1 in dietary PAMP induced APR and lipid responses, we examined responses in mice lacking the IL-1 receptor. Primary hepatocytes isolated from WT mice responded to supernatant of LPS-challenged WT hepatic macrophages by reducing *ApoAI* mRNA, but this response was not observed in hepatocytes isolated from *Il1r1*^−/−^ mice (Figure 10A). Likewise, although a modest SAA protein response to orally-delivered LPS was preserved in HFD-primed *Il1r1*^−/−^ mice *in vivo* (Figure 10B), oral LPS did not significantly alter hepatic *ApoAI* mRNA, serum ApoAI protein, HDL-C or serum cholesterol efflux capacity in *Il1r1*^−/−^ mice (Figures 10C–F). Interestingly, unlike bone-marrow-derived macrophages (BMDM), isolated WT hepatic macrophages released IL-1β protein without requirement for secondary inflammasome stimulus, despite containing lower levels of intracellular IL-1β protein and mRNA (Figures 10G–I). Together, these results suggest that human and mouse hepatocytes are not directly responsive to TLR2 or TLR4 stimulants, but may respond to orally delivered LPS via IL-1β released by activated hepatic macrophages.

## Discussion

Previous studies of the mechanisms connecting microbes, innate immune signaling and cardiometabolic risk have focused largely on the resident intestinal microbiota (36–39). Here, we introduce the concept that the food microbiota may also play a significant role in this relationship, through several findings which are unexpected in light of earlier work.

First, we show that although soluble stimulants of TLR2 and TLR4 are abundant in the gut, they are sequestered mainly in the large intestine, and can be orders of magnitude more abundant in some processed foods than in the healthy ileum. At first glance, this seems counter-intuitive since the human intestinal microbiota comprises >100 trillion bacteria and at least 1 gram of commensal-derived LPS - both of which greatly exceed any quantity that would likely be ingested with food (9). However, we found that the bioactivity of LPS in the human fecal microbiota is far lower (~200-fold) than predicted by numbers of Gram-negative bacteria alone (10). This low activity is likely explained by the fact that the vast majority (>99%) of the Gram-negative bacteria of the human large intestine are members of the Bacteroidetes group (9, 10), which tends to express endotoxin comprising a monophosphoryl, penta-acyl lipid-A that does not stimulate human TLR4/MD2 (40–42). By contrast, proteobacterial species (such as *E. coli*) tend to express a canonical, hexa-acyl bis-phosphorylated lipid A, which is a potent agonist of human TLR4/MD2. The proportionate abundance of proteobacterial 16S rDNA in the tested foods thus helps to explain how these foods can be more immunostimulatory than the mouse fecal microbiota, despite containing much lower total 16S rDNA and bacterial density [up to 10^10^ vs. up to 10^11^ CFU/g, respectively (9, 43)].

Consistent with earlier reports (14, 29), our attempts to measure the absorption of ingested LPS using the limulus assay suggested that the fraction which translocates to blood is extremely small (0.00004%, Figure 4B). However, because circulating LPS is cleared rapidly from the circulation (30), or otherwise masked from detection by limulus enzymes through binding to blood proteins and lipoproteins (44), we sought to confirm this finding using a radiolabel approach. This method revealed that a far greater proportion of ingested LPS is absorbed (~0.4% to liver), than previously estimated using the limulus assay, raising the possibility that LPS absorbed from some dietary sources may exceed the threshold required to trigger hepatic inflammatory signaling.

Our attempts to explore this possibility using an acute model of LPS ingestion were based on the well-established observation of robust acute-phase and lipid responses to intravenously delivered LPS in mice within 24 h (15, 20). However, no such responses were observed when LPS was administered orally to healthy mice. Instead, some form of priming stimulus was found to be necessary to render mice responsive to ingested LPS. Among a number of approaches used to increase responsiveness to LPS (23, 24, 33), HFD-priming was found to be the most effective. This priming effect was not likely due to effects of HFD on gut barrier permeability, since although this has been reported to be increased by some groups (32), we and others (45, 46), did not see this, perhaps due to differences in diet composition.

Instead, it is more likely that priming is related to hepatic macrophage density, which was significantly increased by all of the successful priming regimens (HFD, HCD, *P. acnes* injection and chronic dietary PAMP exposure itself). This view is supported by the observation that systemic responses to ingested LPS were ablated in mice treated with clodronate liposomes. Notably, although clodronate liposome treatment depletes macrophages in diverse tissues systemically, it is likely that hepatic macrophages, rather than macrophages in other tissues, are the predominant mediators of the response to dietary PAMPs in this context. For example, while key APR-inducing cytokines were induced in liver in response to oral LPS, they were not upregulated in other macrophage-rich tissues, including intestinal, adipose and vascular tissues (Figures S1D, S2H; Figures 6I–K). Also, unlike bone-marrow derived macrophages, isolated hepatic macrophages released IL-1β in response to LPS without requirement for priming or secondary stimulus (Figure 8E).

Nevertheless, it should be acknowledged that hepatic macrophages are a highly heterogeneous population, and further work will be required to establish which functional subset is primarily responsible for the observed effects. In particular, it will be helpful to explore whether the response is driven mainly by resident yolk-sac derived Kupffer cells (KCs), or macrophages more recently recruited from the bone marrow, both of which may stain positively for F4/80 and therefore were not distinguished by the present study (47). Evidence suggests that both proliferation of resident KCs and recruitment from blood may drive the accumulation of macrophages during liver injury, and the relative balance of these subsets may help determine heptic responsiveness to PAMPs, since the latter are established to elicit more inflammatory responses to PAMPs than resident KCs (47).

Although we did not see an increase in triglyceride content in the livers of PAMP-fed mice (Figure S2J), our findings may offer some insight to risk factors for non-alcoholic fatty liver disease (NAFLD), since hepatic macrophages are reported to drive the condition in mice (48), and their density is associated with severity of NAFLD in man (49). Markers of low-grade inflammation, particularly those related to TLR-signaling, are also upregulated in livers of obese patients compared to healthy controls (50), and the condition is more common in subjects consuming the Western diet (which is relatively high in PAMPs) compared to the Mediterranean diet (which is based more on low PAMP foods) (51).

The induction of inflammatory cytokines and APR signaling in the liver by ingested PAMPs could also be of potential relevance to cardiovascular risk. Experimentally induced APR-signaling, restricted specifically to hepatocytes, has been shown to accelerate atherosclerosis in *Apoe*^−/−^ mice (52). Several lines of evidence also suggest that serum efflux capacity, and the RCT pathway more generally, may be key regulators of cardiovascular risk. For example, serum RCT capacity is inversely associated with CAD risk in man (53), and protective roles have been demonstrated for RCT and ApoAI, in murine models of atherosclerosis (54–56). It will be interesting to explore in further studies whether dietary PAMP intake modifies atherogenesis in mice, or serum RCT function in man.

**Figure 11:**
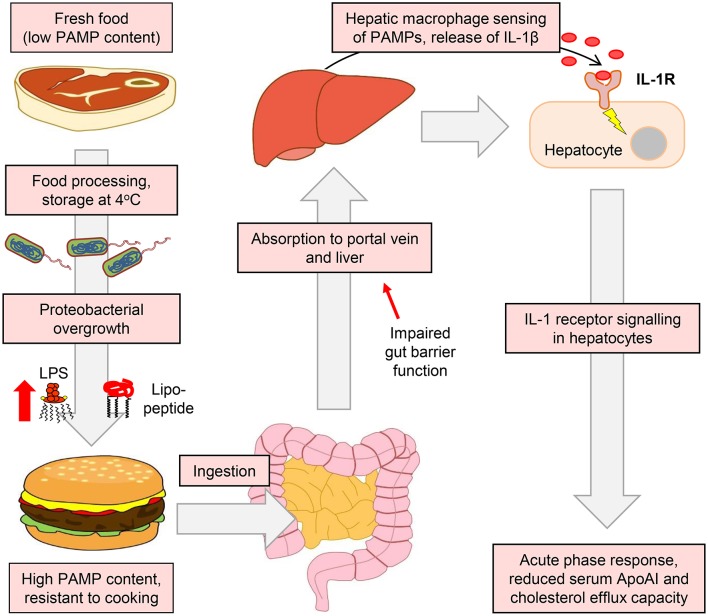
Suggested model for the accumulation of PAMPs in foods and their impact on APR and RCT mediators. The enrichment of the food microbiota in proteobacterial species and their subsequent overgrowth can occur in meat and certain other food products when finely chopped and stored at or above refrigeration temperature. This leads to the accumulation of pro-inflammatory bacterial lipopeptides and lipopolysaccharides, which retain their TLR2 and TLR4-stimulating properties even after cooking has killed remaining viable bacteria (26). These TLR-stimulants may then be absorbed, particularly when intestinal barrier function is impaired, to reach the portal circulation and the liver. Although hepatocytes are insensitive to these stimulants, they may be detected by hepatic macrophages, which secrete IL-1β and other inflammatory cytokines to trigger the APR programme in hepatocytes. This results in reduced serum levels of ApoAI, which is a key mediator of RCT and atherogenesis in mice (55). Modulation of the food microbiota therefore represents a novel potential target for the modulation of systemic inflammatory markers and cholesterol metabolism.

Our results also suggest a key role for IL-1 signaling in the ApoAI and efflux responses to ingested LPS. For example, IL-1β-neutralizing antibody blunted the suppression of hepatocyte *ApoAI* expression by the conditioned media of stimulated macrophages (Figure 9G), and responses to ingested LPS were also blunted or absent in *Il1r1*^−/−^ mice (Figures 10A–E). Together, these findings support a model in which hepatic macrophage-dependent sensing of food-borne PAMPs triggers a mild APR programme in hepatocytes, driven largely via release of IL-1β, resulting in the downregulation of ApoAI synthesis, reduced HDL-C and impaired RCT to serum (Figure 11).

These findings offer useful new insight into the emerging understanding that IL-1 plays a key role in the development of both NAFLD (48) and cardiovascular disease (1). For example, the recent CANTOS trial showed that IL-1 neutralization with Canakinumab significantly reduced both APR markers and cardiovascular event rate in patients with a history of myocardial infarction (1). However, a key question raised by these studies is what might be the source and nature of the triggers for the IL-1 production underpinning these findings. The present study suggests that dietary PAMPs may be a plausible candidate for IL-1 stimulation in this context, and that IL-1 mediated reduction in RCT mediators may be a mechanism for the protective activity of Canakinumab.

It should be acknowledged that TLR2 and TLR4 stimulants are well-established to trigger systemic inflammation and dyslipidaemia when delivered at high doses intravenously (15, 20). However, it has been generally assumed to date that these stimulants should not induce similar effects when ingested. This view is based on the assumption that foods should contain a far lower PAMP content than the intestinal microbiota, the observation of no obvious deleterious phenotype (inflammatory or otherwise) following acute oral administration of LPS or the TLR2-stimulant Pam_3_CSK_4_ to unprimed animal models (12, 13), the well-recognized abundance of TLR2- and TLR4-stimulants produced by the commensal microbiota of the large intestine (10) and the presumed low absorbance of ingested PAMPs (14, 29). The present study provides evidence to counter each of these assumptions for the reasons outlined above.

If present in man, this pathway also has potential to shed light on several key questions in dietary epidemiology. For example, it remains unclear why the consumption of processed meats increases risk of CAD and type II diabetes to a greater degree than their unprocessed equivalents, which are otherwise identical in macronutrient content (57–59). Likewise, the mechanisms connecting processed food consumption with systemic inflammatory markers in cross-sectional studies are poorly defined (2, 60, 61). The present findings suggest that many processed foods, particularly those based on minced meats, may contain a higher PAMP content than their fresh or unchopped equivalents, due to historical overgrowth of proteobacteria (7). The chronic consumption of such foods could therefore impact on markers of systemic inflammation and lipid metabolism, via the mechanisms outlined above, as supported by the results of our earlier dietary trials in human volunteers (8). Notably, our findings do not suggest that commonly used probiotic or fermentative organisms should induce similar effects, since almost all such bacteria are Gram-positive, and as such do not produce LPS or shed abundant stimulants of other TLRs into their environment [Figure 2F and (26)].

In summary, we show that although the large intestine harbors a large microbial flora, molecules released by the proteobacteria-dominated microbiota of some processed foods may have potential to dysregulate inflammatory and metabolic pathways systemically when ingested. This mechanism helps to explain the long-standing connection between processed meat intake and metabolic disease, and introduces the food spoilage microbiota as an unexpected potential mediator of cardiovascular risk. Further studies are warranted to test whether cholesterol metabolism may be modified through simple interventions targeting the food microbiota, or their pro-inflammatory products.

## Data Availability

The datasets generated for this study are available on request to the corresponding author.

## Ethics Statement

All mouse experiments were conducted according to Home Office guidelines and with institutional and Home Office approval (PPL60/4332). Human blood samples were collected from informed, consenting healthy volunteers according to the guidelines laid down in the Declaration of Helsinki and with approval from the University of Leicester College of Medicine Research Ethics Committee.

## Author Contributions

CE and TF performed experiments and analyzed results. CE and CS secured funding for and managed the project. CE planned the study and wrote the paper.

### Conflict of Interest Statement

The authors declare that the research was conducted in the absence of any commercial or financial relationships that could be construed as a potential conflict of interest.

## Abbreviations

ApoAI: Apolipoprotein AI
APR: Acute phase response
CAD: Coronary artery disease
iEDAP: D-gamma-Glu-meso-diaminopimelic acid
GAPDH: Glyceraldehyde 3-phosphate dehydrogenase
HBSS: Hank's balanced salt solution
HOMA-IR: Homeostatic model assessment of insulin resistance
KC: Kupffer cell
LAL: Limulus amoebocyte lysate
LPS: Lipopolysaccharide
NAFLD: Non-alcoholic fatty liver disease
NOD: Nucleotide-binding oligomerization domain-containing protein
OxPAPC: Oxidized palmitoyl arachidonyl phosphatidyl choline
PAMP: Pathogen-associated molecular pattern
PBMC: Peripheral blood mononuclear cells
RCT: Reverse cholesterol transport
rDNA: Bacterial 16S ribosome gene DNA
SAA: Serum amyloid A
SPF: Specific pathogen free
TLR: Toll-like receptor
TNF: Tumor necrosis factor.
