# Supplementary material for: Dietary Toll-Like Receptor Stimulants Promote Hepatic Inflammation and Impair Reverse Cholesterol Transport in Mice via Macrophage-Dependent Interleukin-1 Production
Source: Front Immunol. 2019 Jun 20;10:1404. doi: 10.3389/fimmu.2019.01404 (PMC6611433; doi:10.3389/fimmu.2019.01404)
Supplement: Supplementary file 1 [file Data_Sheet_1.PDF]

Supplemental Figures

Dietary Toll-like Receptor Stimulants Promote Hepatic Inflammation and Impair Reverse Cholesterol Transport in Mice via Macrophage-Dependent Interleukin-1 Production

Tola A Faraj (PhD), Cordula Stover (Dr med, PhD), Clett Erridge (PhD)

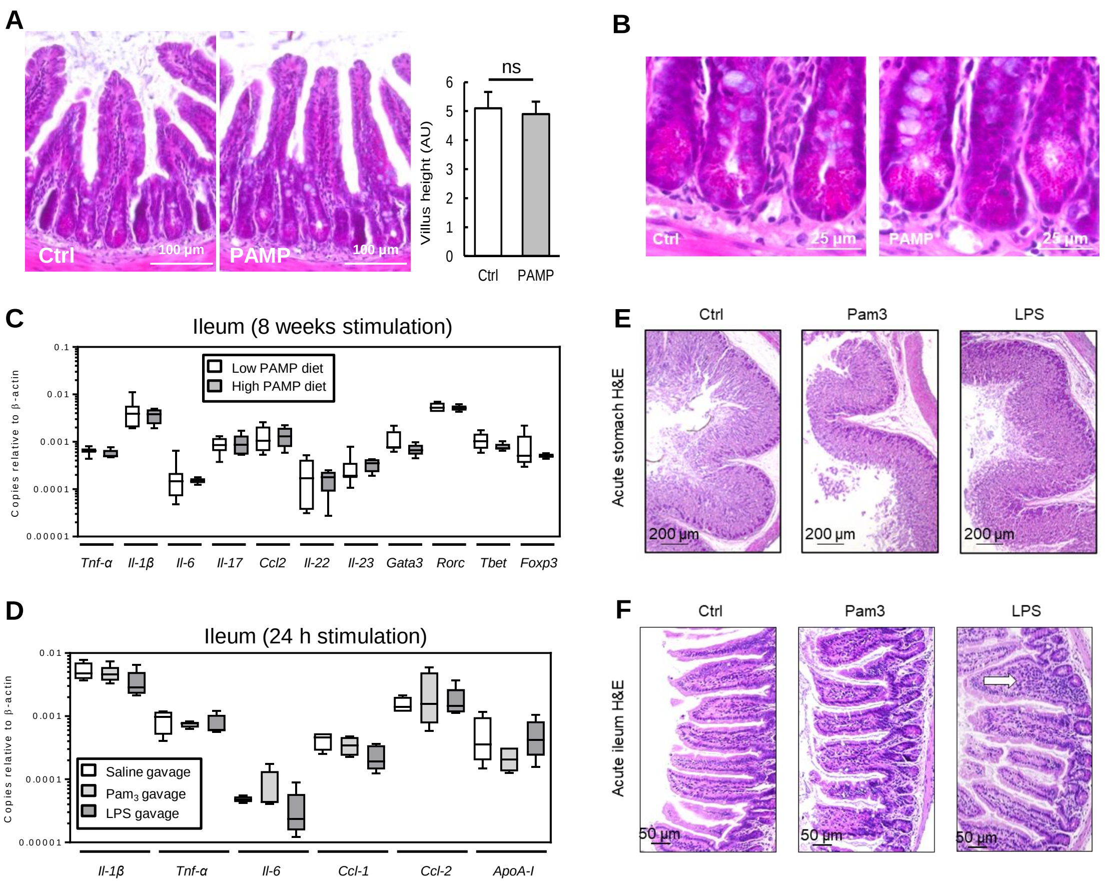

Figure S1: Intestinal inflammatory markers are not induced by acute or chronic dietary PAMP intake in mice

WT mice (n=8/gp) were given normal chow and drinking water supplemented (PAMP) or not (Ctrl), with 100 µg/ml *E. coli* LPS, 1 µg/ml Pam_3CSK_4 and 1 µg/ml iEDAP for 8 weeks. **(A,B)** There was no histological evidence of inflammation in ileum of PAMP-fed mice, as supported by no significant change in villus height or paneth cell degranulation. **(C)** There were no significant differences in abundance of mRNA for key intestinal innate and adaptive immune cytokines, nor markers of T-helper lymphocyte subset polarisation in mice chronically exposed to dietary PAMPs. **(D)** There were also no significant differences in abundance of mRNA for key intestinal inflammatory cytokines, or apolipoprotein (Apo)-AI, nor histological evidence of inflammation in stomach **(E)** or ileum **(F)**, 24 h after oral gavage with 200 µl saline alone (Ctrl), or 1 mg *E. coli* LPS, or 1 mg Pam_3CSK_4 (n=5/gp), aside from very occasional villi (example shown by white arrow) in LPS-treated mice. Error bars shown are SEM. P-values vs control condition, T-test and ANOVA with Dunnett's test.

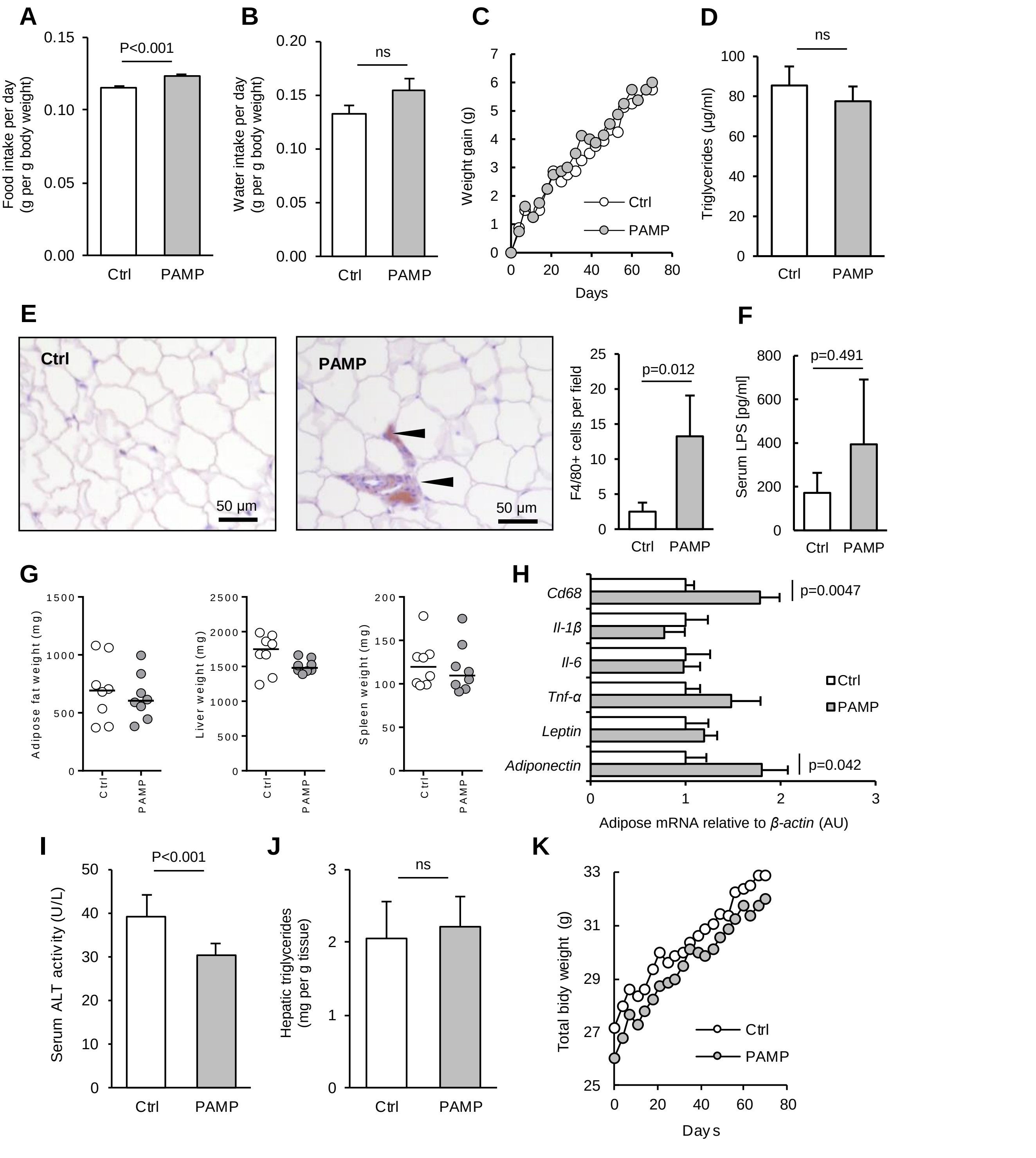

Figure S2: Chronic dietary PAMP intake increases adipose macrophage number, but not activation

WT mice (n=8/gp) were given normal chow and drinking water supplemented (PAMP) or not (Ctrl), with 100 µg/ml *E. coli* LPS, 1 µg/ml Pam_3CSK_4 and 1 µg/ml iEDAP for 8 weeks. **(A,B)** Food and water intake in control and PAMP-fed mice. **(C,D)** PAMP intake did not change weight gain or plasma triglycerides. **(E)** PAMP intake caused a ~5-fold increase in abundance of F4/80+ staining cells (macrophages) in abdominal adipose tissue. **(F)** Serum LPS levels measured using the limulus assay. **(G)** PAMP intake did not affect weight of abdominal adipose tissue, liver or spleen. **(H)** Expression of key inflammatory markers of macrophage activation was not increased in adipose tissue of PAMP-fed mice. **(I)** Serum alanine aminotransferase (ALT) activity in control and PAMP-fed mice. **(J)** Liver triglyceride content of control and PAMP-fed mice. **(K)** Mean total body weights of each group of mice. P-values vs control condition. Error bars shown are SEM.

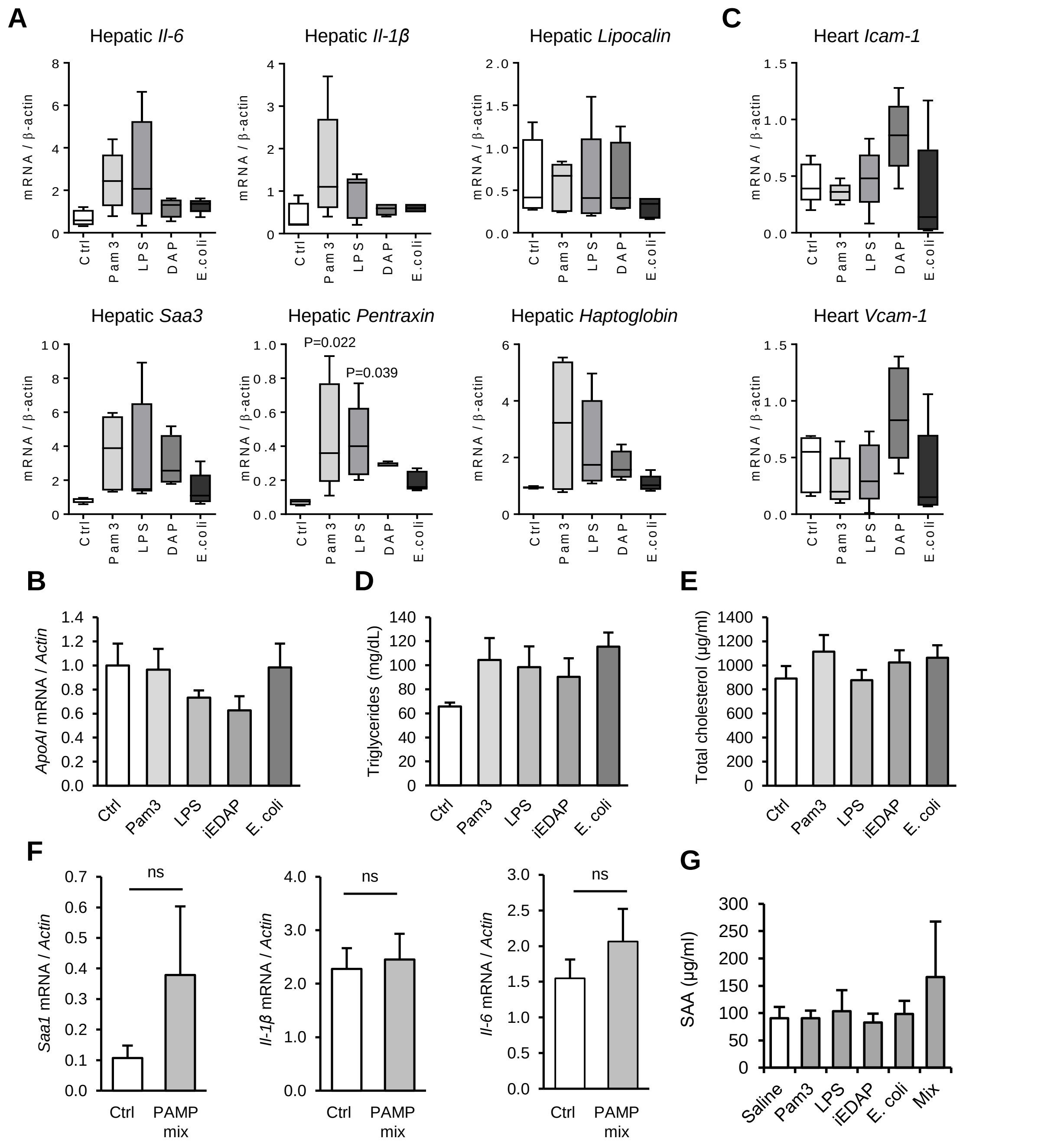

Figure S3: A single oral gavage with TLR2, TLR4 or NOD1 stimulants fails to reproducibly alter serum lipid levels or inflammatory markers in liver and heart

WT mice fed normal chow (n=5/gp) were orally gavaged with 1 mg Pam_3CSK_4, 1 mg *E. coli* LPS, 1 mg iEDAP (a component of peptidoglycan which stimulates NOD1), or 2x1010 washed heat-killed *E. coli* cells (mixed PAMP stimulus). Tissues and sera were harvested 24 h after gavage. **(A,B)** Hepatic inflammatory and acute phase response (APR) marker mRNAs were not reproducibly increased. **(C)** There was no significant induction of the inflammatory markers vascular cell adhesion molecule-1 (VCAM-1) or intercellular adhesion molecule-1 (ICAM-1) in heart following the same treatment. **(D,E)** Serum triglyceride and cholesterol levels were not reproducibly increased by a single exposure to orally delivered tested stimulants. **(F)** Hepatic inflammatory markers in WT mice (n=5) orally gavaged with a mixture of 1 mg Pam_3CSK_4, 2 mg *E. coli* LPS and 1 mg iEDAP. **(G)** Plasma serum amyloid A (SAA) protein 24 h after oral gavage with saline alone (Ctrl), iEDAP, Pam_3CSK_4, *E. coli* LPS or a mixture of these PAMPs (n=5/gp). Error bars shown are SEM. P-values vs control condition, ANOVA with Dunnett's test.
